# Tumour microenvironment and HER2-low status dictate response and resistance to anthracycline-taxane chemotherapy in premenopausal TNBC: a retrospective multicohort study

**DOI:** 10.3389/fphar.2026.1752328

**Published:** 2026-02-09

**Authors:** Shuanglong Cai, Shaohong Yu, Quan Zhou, Fangyuan Kuang, Lingyan Rao, Zhen Fang, Xiaoxin Zheng, Yong Shi, Jingdan Li

**Affiliations:** 1 Department of Thyroid and Breast Surgery, Comprehensive Breast Health Center, The First Affiliated Hospital of Lishui University, Lishui People’s Hospital, Lishui, Zhejiang, China; 2 Peking Union Medical College Hospital, Peking Union Medical College and Chinese Academy of Medical Sciences, Beijing, China; 3 Department of Gynecology, Fujian Provincial Hospital, Fuzhou University Affiliated Provincial Hospital, Fuzhou, Fujian, China; 4 Department of Cardiology, Fuzhou University Affiliated Provincial Hospital, Shengli Clinical Medical College of Fujian Medical University, Fujian Provincial Hospital, Fuzhou, Fujian, China; 5 Fujian Provincial Key Laboratory of Cardiovascular Disease, Fujian Cardiovascular Institute, Fujian Provincial Center for Geriatrics, Fujian Clinical Medical Research Center for Cardiovascular Diseases, Fuzhou, Fujian, China; 6 Fujian Heart Failure Center Alliance, Fuzhou, Fujian, China

**Keywords:** anthracycline-taxanechemotherapy, biomarkers, chemoresistance, HER2-low, premenopausal triple-negative breast cancer, stromal tumor infiltrating lymphocytes (sTILs)

## Abstract

**Background:**

Premenopausal women with triple-negative breast cancer (TNBC) exhibit considerable heterogeneity in their response to standard anthracycline and taxane-based chemotherapy, yet the underlying mechanisms remain poorly understood. We aimed to investigate the combined role of the tumour immune microenvironment and HER2-low status in predicting chemosensitivity and intrinsic resistance in this specific population.

**Methods:**

We retrospectively analysed data from 767 premenopausal patients with TNBC across two Chinese medical centres. All patients underwent primary surgery followed by adjuvant chemotherapy based on anthracyclines and taxanes. Patients were randomly assigned to training and internal validation cohorts. Independent predictors of DFS were identified using multivariable Cox proportional hazards regression, and a nomogram was constructed accordingly. The model’s discrimination was assessed using the concordance index (C-index) and time-dependent receiver operating characteristic (ROC) curve analysis, while calibration was evaluated with calibration curves.

**Results:**

Multivariable analysis identified lower stromal tumour-infiltrating lymphocyte (sTIL) expression levels, and HER2 IHC 2+/FISH-negative status as independent factors associated with poorer DFS, besides that T3/T4 staging, higher N staging. A nomogram integrating these four variables demonstrated excellent predictive accuracy for DFS, with a C-index of 0.862 in the training set and 0.861 in the validation set. The area under the ROC curve (AUC) for predicting 3-year DFS was 0.907 and 0.908 in the training and validation sets, respectively.

**Conclusion:**

Our findings reveal that an immune-poor tumour microenvironment and HER2-low biology are key, complementary determinants of intrinsic resistance to standard chemotherapy in premenopausal TNBC. These readily available biomarkers provide a mechanistic rationale for patient stratification, suggesting that sTIL-low tumours might benefit from immunomodulatory strategies, while HER2-low tumours represent a candidate population for novel antibody-drug conjugates. This paradigm shift from empirical to biomarker-informed therapy could help overcome chemoresistance in this high-risk group.

## 1 Introduction

Breast cancer remains the most commonly diagnosed malignancy and the leading cause of cancer-related mortality among women worldwide (Bray et al., 2024). Triple-negative breast cancer (TNBC), characterized by the absence of estrogen receptor, progesterone receptor, and HER2 amplification, represents a particularly aggressive subtype with limited therapeutic options and a heightened propensity for early recurrence (Bianchini et al., 2016; Foulkes et al., 2010). Within this heterogeneous disease, premenopausal patients often exhibit distinct clinicopathological and molecular features, including enriched immunogenic signatures and homologous recombination deficiency, which may influence both tumour behaviour and therapeutic response (Azim et al., 2012; Bareche et al., 2018).

Anthracycline and taxane-based chemotherapy remains the cornerstone of adjuvant treatment for early-stage TNBC (Liedtke et al., 2008). However, intrinsic and acquired chemoresistance significantly undermines its efficacy, leading to disparate clinical outcomes (Gonzalez-Angulo et al., 2011; Wahba and El-Hadaad, 2015). The mechanisms underlying chemoresistance are multifactorial, encompassing tumour-intrinsic factors such as apoptotic evasion, enhanced DNA damage repair, and cancer stem cell persistence, as well as microenvironmental influences including immune contexture and stromal composition (Holohan et al., 2013; Shibue and Weinberg, 2017; Gottesman, 2002).

Emerging evidence underscores the prognostic significance of stromal tumour-infiltrating lymphocytes (sTILs) in TNBC, where higher densities correlate with improved pathological complete response and survival following chemotherapy (M Geurts et al., 2024; Salgado et al., 2015). Concurrently, The biological and clinical significance of HER2-low breast cancer, defined as immunohistochemistry (IHC) 1+ or IHC 2+ with negative *in situ* hybridization (FISH), has recently come into focus. Supported by evidence from clinical trials of novel anti-HER2 antibody–drug conjugates, this subset has emerged as a therapeutically actionable entity (Saura et al., 2025; Modi et al., 2025). Growing preliminary evidence further suggests that HER2-low tumours may represent a distinct biological subtype with unique therapeutic vulnerabilities (Tarantino et al., 2020; Agostinetto et al., 2021). Recent studies, including those by Tarantino P and Schettini F et al., have begun to delineate the underlying biology of HER2-low breast cancer across subtypes, including triple-negative breast cancer (Tarantino et al., 2023; Schettini et al., 2025).

Despite these advances, validated biomarkers for stratifying chemosensitivity in premenopausal TNBC remain scarce. Most existing prognostic tools neither account for the unique biology of this demographic nor integrate readily accessible biomarkers reflective of tumour-immune interplay (Sparano et al., 2018; Parker et al., 2009). Therefore, we aimed to develop and validate a clinically applicable nomogram that incorporates key clinicopathological and microenvironmental variables-specifically sTIL density and HER2-low status-to predict disease-free survival (DFS) in premenopausal women with TNBC treated with anthracycline-taxane chemotherapy.

## 2 Methods

### 2.1 Study design and patient cohort

**FIGURE 1:**
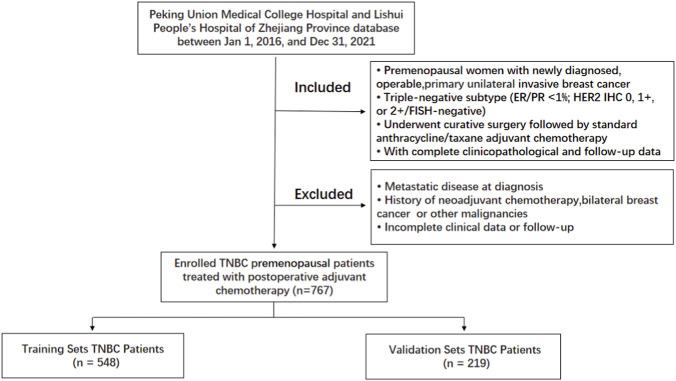
Patient selection flowchart for this study.

This retrospective, multicentre cohort study consecutively enrolled 767 female patients with TNBC from Peking Union Medical College Hospital and Lishui People’s Hospital of Zhejiang Province between 1 Jan 2016, and 31 Dec 2021 (Figure 1).

Inclusion Criteria: (1) Histopathologically confirmed invasive breast carcinoma; (2) Negative for estrogen receptor, progesterone receptor, and HER2 (defined as ER/PR <1% positive cells, HER2 IHC 0 or 1+, or 2+ with negative FISH); (3) Premenopausal status at diagnosis; (4) Underwent curative-intent surgery (modified radical mastectomy or breast-conserving surgery) at initial diagnosis; (5) Received adjuvant chemotherapy based on anthracyclines and taxanes (sequential or combination regimens) postoperatively; (6) Had complete clinical, pathological, and follow-up data.

Exclusion Criteria: (1) Bilateral breast cancer or concurrent other malignancies; (2) History of neoadjuvant chemotherapy; (3) Presence of distant metastasis at diagnosis; (4) Incomplete follow-up data.

### 2.2 Variable definition and data collection

Collected variables included: age at diagnosis, family history of breast cancer, surgical approach, postoperative pathological T staging, postoperative pathological N staging, tumor grade, lymph-vascular invasion, sTIL expression levels, Her2 expression levels, Ki-67 index, CK5/6 expression, EGFR expression, and receipt of radiotherapy.

HER2 Status Assessment: HER2 protein expression was evaluated by IHC, and equivocal cases (IHC 2+) were reflexively tested by FISH according to the American Society of Clinical Oncology/College of American Pathologists (ASCO/CAP) guidelines (Wolff et al., 2023). To ensure consistency and mitigate inter-institutional variability-a known challenge in HER2-low scoring—all IHC and FISH results underwent a centralized review by two experienced breast pathologists who were blinded to patient outcomes. HER2-low status was defined according to the standard definition as IHC 1+ or IHC 2+/FISH-negative. For primary and sensitivity analyses, patients were categorized into three groups: HER2 IHC 0, HER2 IHC 1+, and HER2 IHC 2+/FISH-negative.

sTIL Assessment: sTILs were evaluated by two experienced pathologists blinded to clinical outcomes, according to international guidelines. sTILs were defined as the percentage of tumour stroma area occupied by mononuclear inflammatory cells (lymphocytes and plasma cells). sTIL density was categorised into three groups: low (≤10%), intermediate (10%–40%), and high (>40%).

### 2.3 Follow-up strategy

Follow-up started from the date of initial breast cancer diagnosis and ended on 1 June 2025. Follow-up methods included outpatient visits and telephone interviews. Data collected encompassed: local or regional recurrence, distant metastasis, time to first recurrence or metastasis, site of first recurrence or metastasis, and vital status at the last follow-up.

Disease-free survival (DFS) was defined as the time from surgery date to the first occurrence of any of the following events: local recurrence, regional recurrence, distant metastasis, contralateral breast cancer, or death from any cause.

### 2.4 Statistical analysis

Analyses were performed using R software (version 4.3.1). The patient cohort was randomly split into a training set (70%) and an internal validation set (30%). Categorical variables are presented as numbers (percentages) and compared using the χ^2^ test or Fisher’s exact test. Continuous variables are presented as mean ± standard deviation or median (interquartile range) based on their distribution and compared using the t-test or Mann-Whitney U test.

In the training set, univariable Cox regression analysis (P < 0.05) was used to screen prognostic factors associated with DFS. Significant variables were subsequently included in a multivariable Cox proportional hazards regression model to identify independent prognostic factors. Results are presented as hazard ratios (HRs) with 95% confidence intervals (CIs).

Based on the final independent prognostic factors, a nomogram for predicting 1-year, 3-year, and 5-year DFS probabilities was constructed using the rms package. The model’s discrimination was assessed using the C-index and time-dependent AUC analysis. Calibration curves were plotted to evaluate the agreement between predicted probabilities and observed outcomes (assessed by the Kaplan-Meier method). For key prognostic variables, Kaplan-Meier survival curves were generated, and differences between groups were compared using the log-rank test.

All tests were two-sided, and a P value <0.05 was considered statistically significant.

## 3 Results

### 3.1 Patient characteristics and follow-up

The final cohort comprised 767 premenopausal TNBC patients with a median follow-up of 76.0 months. The median age was 49.0 years. The cohort was randomly divided into training (n = 548) and internal validation (n = 219) sets. Baseline clinicopathological characteristics were largely balanced between the training and validation sets, except for family history, postoperative pathological T staging, Ki-67 index, and EGFR status (all P < 0.05, Table 1). All other variables showed no significant differences (all P > 0.05, Table 1). In the training set, 420 DFS events and 128 deaths were recorded over a median follow-up of 73.7 months; the validation set experienced 170 DFS events and 49 deaths over 75.4 months.

**TABLE 1:** The clinical-pathological characteristics of TNBC patients in both the training and validation sets.

Variables	Training sets (n = 548)	Validation sets (n = 219)	P Value
Age at diagnosis	​	​	0.531
<50 years	451 (82.30%)	176 (80.37%)	​
≥50 years	97 (17.70%)	43 (19.63%)	​
Family history	​	​	0.020
No	469 (85.58%)	201 (91.78%)	​
Yes	79 (14.42%)	18 (8.22%)	​
Surgical approach	​	​	0.396
Radical surgery	412 (75.18%)	171 (78.08%)	​
Breast-conserving surgery	136 (24.82%)	48 (21.92%)	​
Postoperative pathological T staging	​	​	<0.001
T1	172 (31.39%)	91 (41.55%)	​
T2	264 (48.18%)	65 (29.68%)	​
T3+T4	112 (20.44%)	63 (28.77%)	​
Postoperative pathological N staging	​	​	0.183
N0	391 (71.35%)	153 (69.86%)	​
N1	109 (19.89%)	47 (21.46%)	​
N2	27 (4.93%)	16 (7.31%)	​
N3	21 (3.83%)	3 (1.37%)	​
Tumor grade	​	​	0.170
G1+G2	347 (63.32%)	127 (57.99%)	​
G3	201 (36.68%)	92 (42.01%)	​
Lymph-vascular invasion	​	​	0.088
No	283 (51.64%)	128 (58.45%)	​
Yes	265 (48.36%)	91 (41.55%)	​
sTIL expression levels	​	​	0.427
Low	117 (21.35%)	38 (17.35%)	​
Intermediate	162 (29.56%)	71 (32.42%)	​
High	269 (49.09%)	110 (50.23%)	​
Her2 expression levels	​	​	0.522
IHC 0	218 (39.78%)	95 (43.38%)	​
IHC 1+	169 (30.84%)	59 (26.94%)	​
IHC 2+/Fish-	161 (29.38%)	65 (29.68%)	​
Ki67	​	​	0.005
Ki67 ≤ 20	22 (4.01%)	20 (9.13%)	​
Ki67 > 20	526 (95.99%)	199 (90.87%)	​
CK5/6	​	​	0.995
Negative	115 (20.99%)	46 (21.00%)	​
Positive	433 (79.01%)	173 (79.00%)	​
EGFR	​	​	0.031
Negative	91 (16.61%)	51 (23.29%)	​
Positive	457 (83.39%)	168 (76.71%)	​
Radiation therapy status	​	​	0.731
No	442 (80.66%)	179 (81.74%)	​
Yes	106 (19.34%)	40 (18.26%)	​

Family history, HBOC-related cancer history.

sTIL, stromal tumor-infiltrating lymphocytes; IHC, mmunohistochemistry.

CK5/6, cytokeratin 5/6; HER2, human epidermal growth factor receptor 2.

FISH, fluorescence *in situ* hybridization; EGFR, epidermal growth factor receptor.

### 3.2 Prognostic nomogram for disease-free survival

**FIGURE 2:**
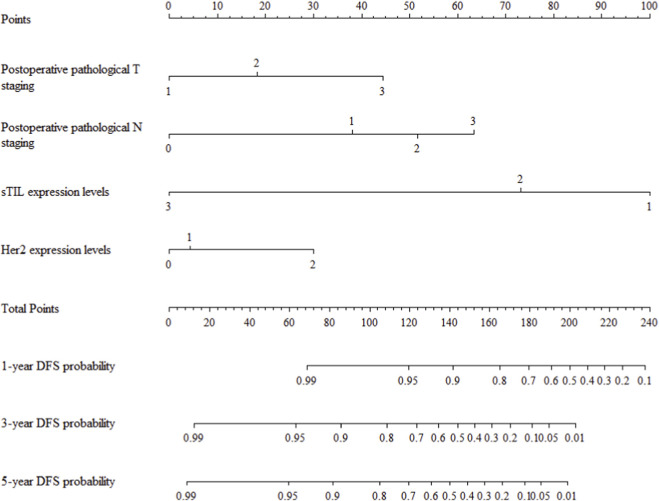
Prognostic nomogram for predicting disease-free survival time of premenopausal TNBC patients.

**FIGURE 3:**
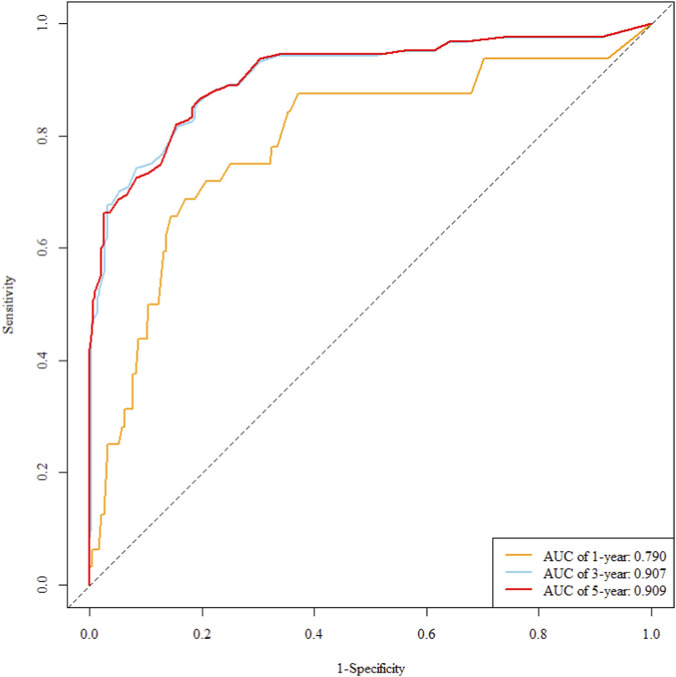
Roc curve of the training set.

**FIGURE 4:**
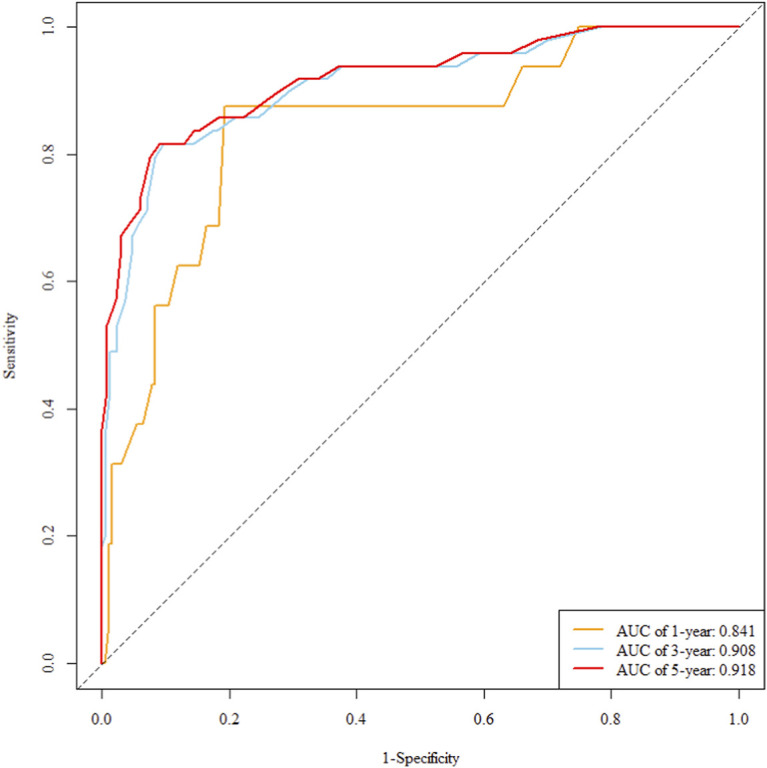
Roc curve of the validation set.

**FIGURE 5:**
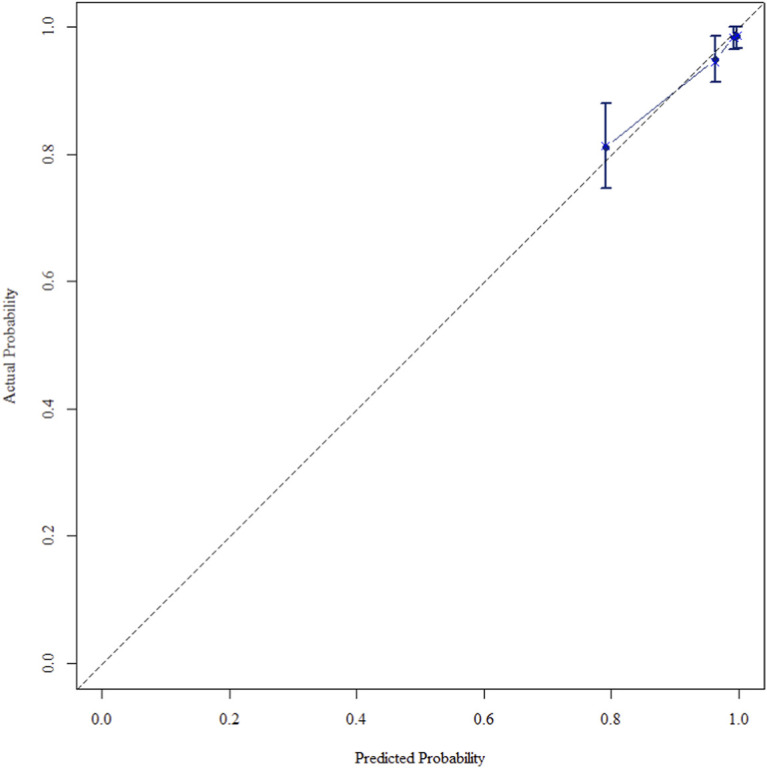
Calibration curve of the training set at 1 Year.

**FIGURE 6:**
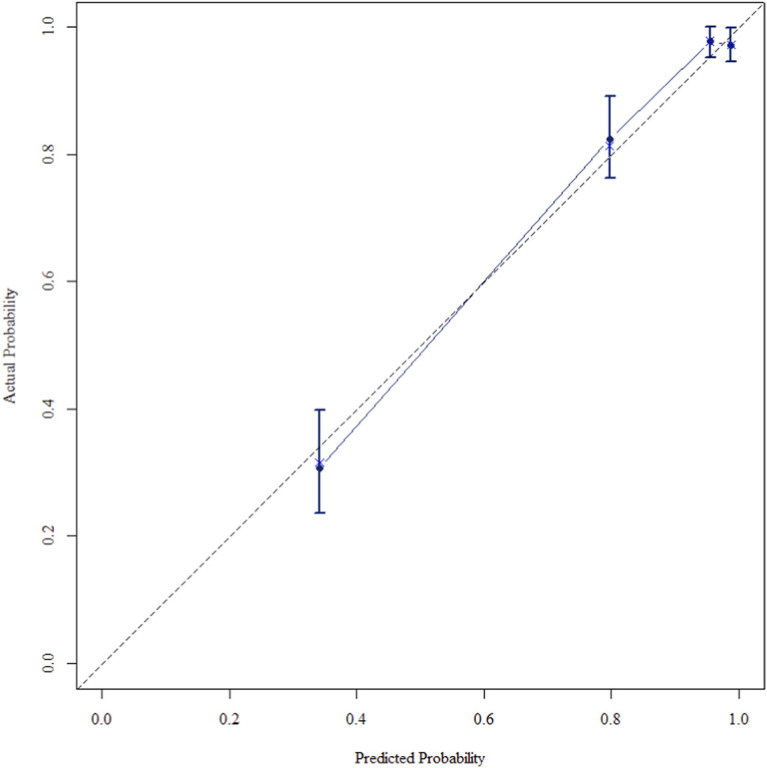
Calibration curve of the training set at 3 Years.

**FIGURE 7:**
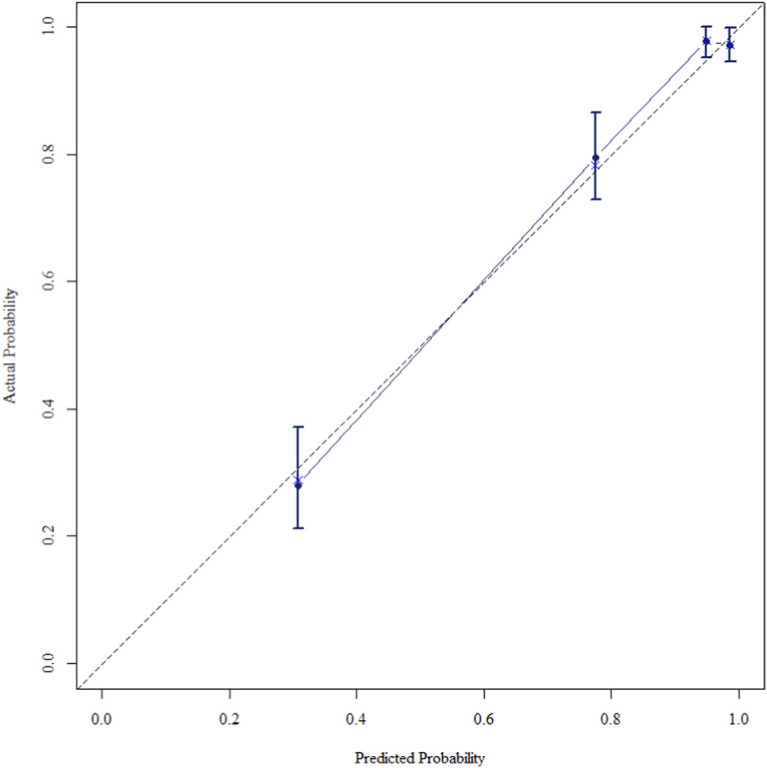
Calibration curve of the training set at 5 Years.

**FIGURE 8:**
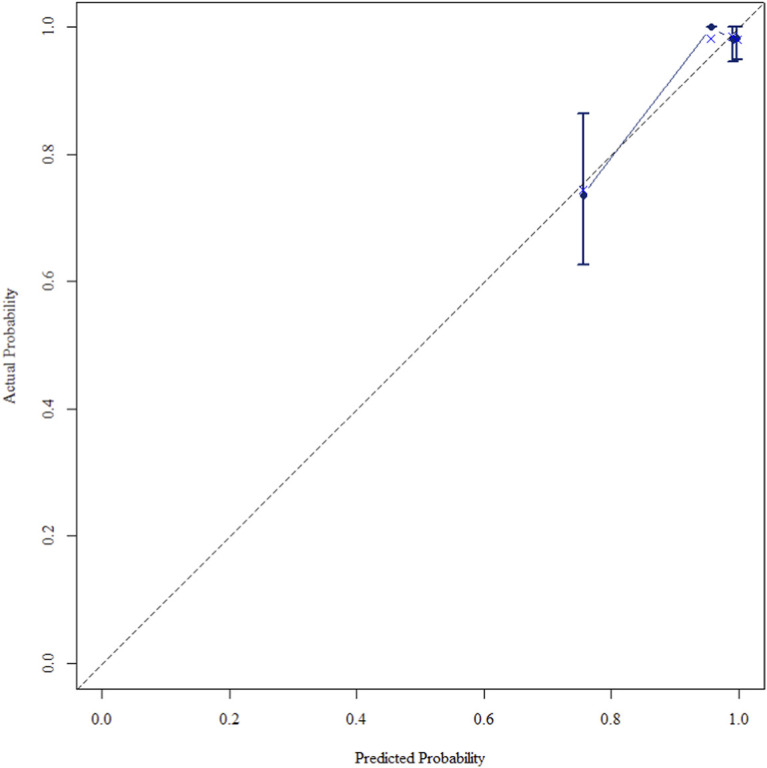
Calibration curve of the validation set at 1 Years.

**FIGURE 9:**
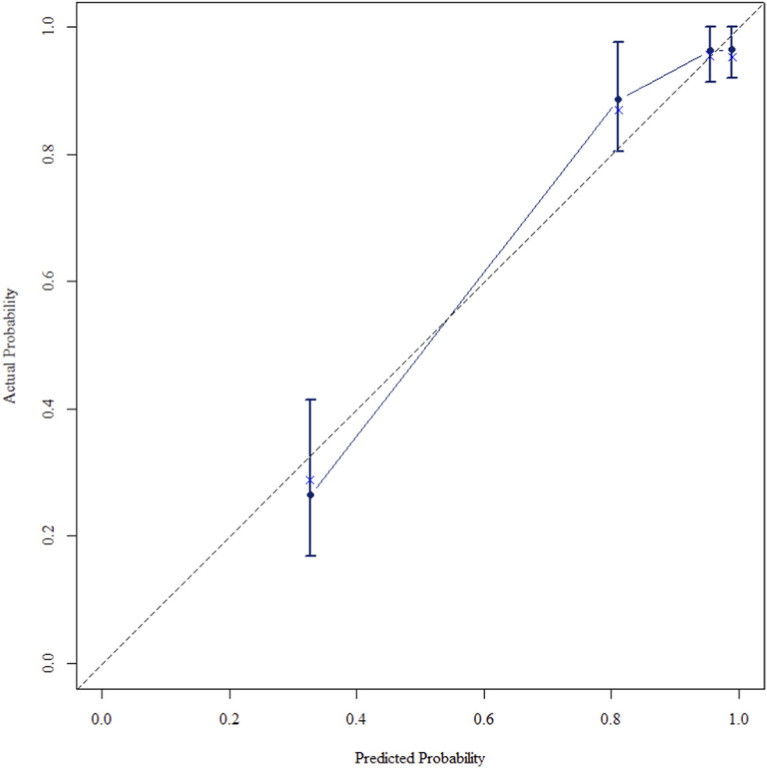
Calibration curve of the validation set at 3 Years.

**FIGURE 10:**
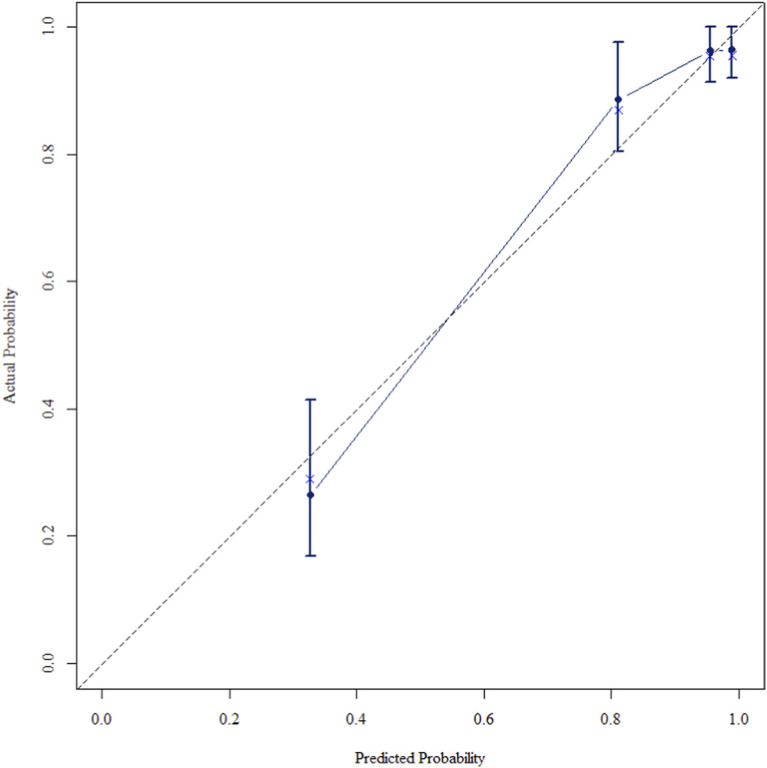
Calibration curve of the validation set at 5 Years.

**FIGURE 11:**
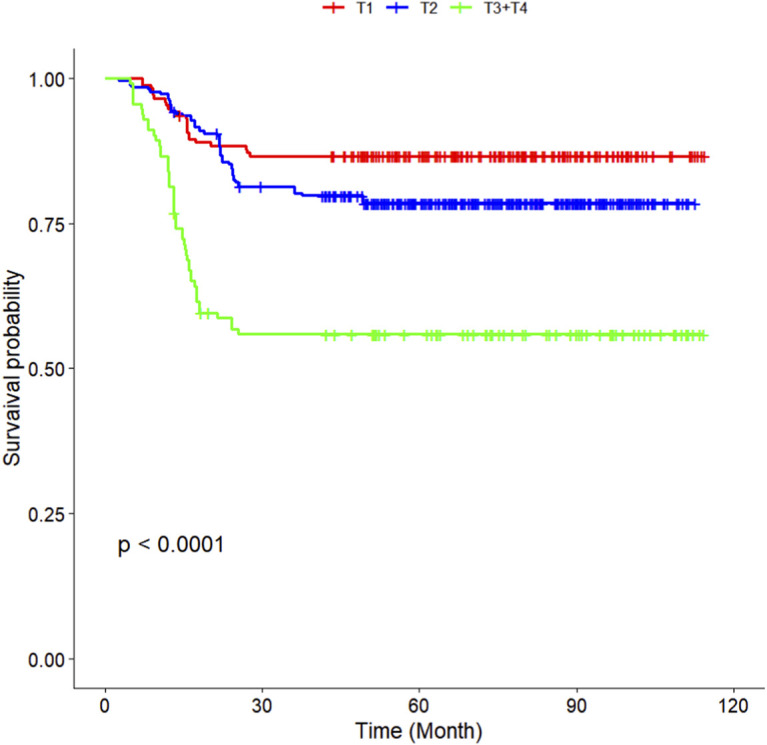
KM curves stratified by T staging in the Training Set.

**FIGURE 12:**
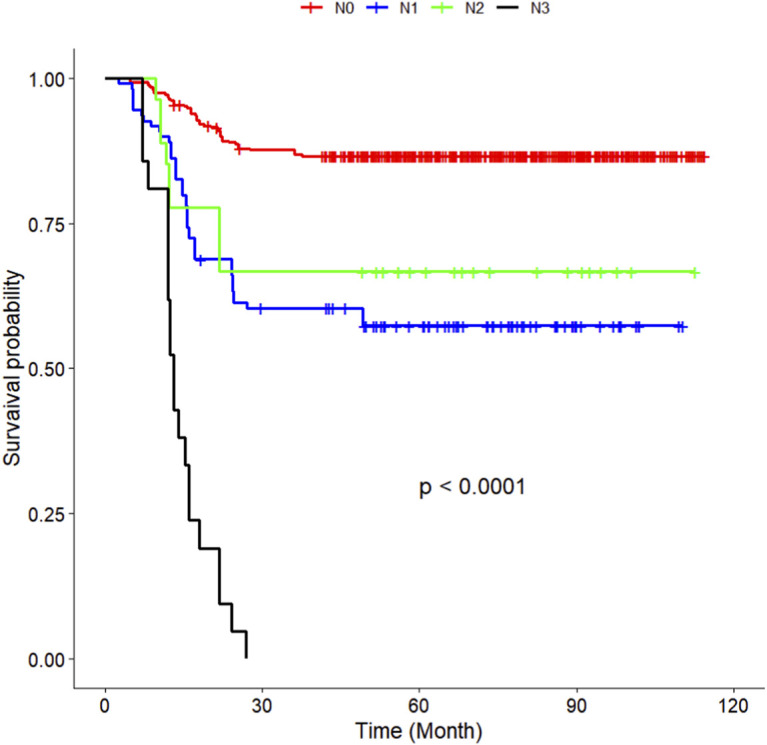
KM curves stratified by N staging in the Training Set.

**FIGURE 13:**
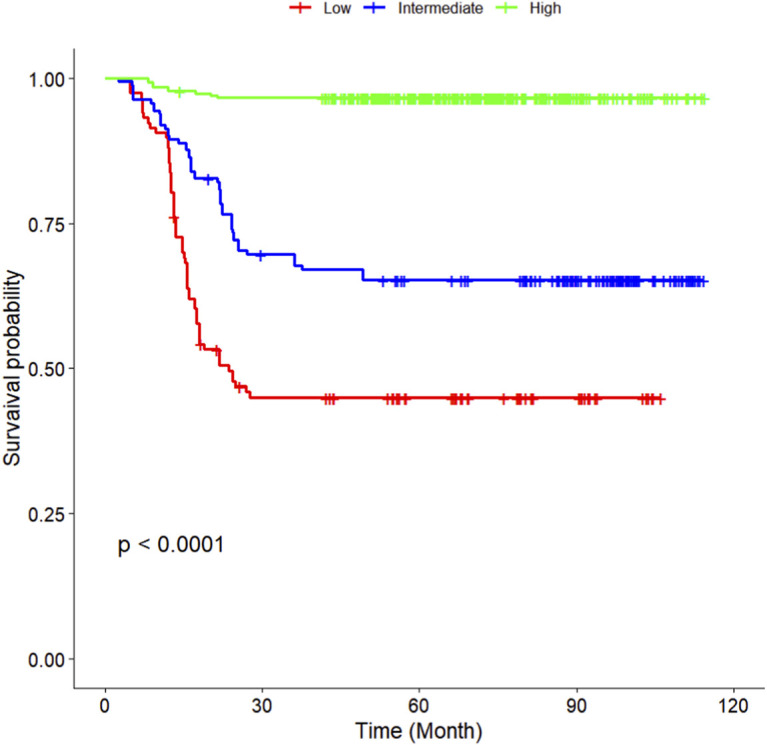
KM curves stratified by sTIL expression levels in the Training Set.

**FIGURE 14:**
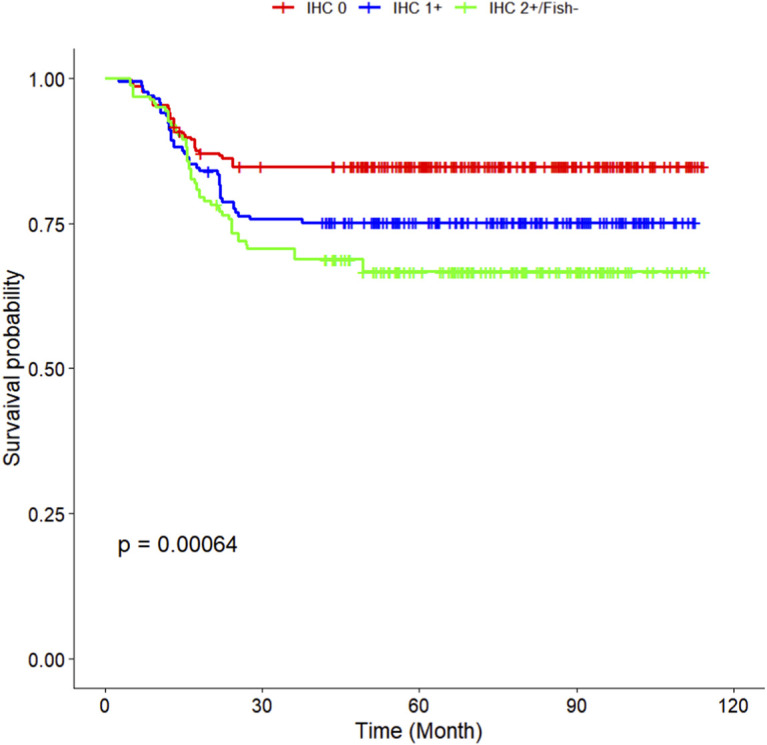
KM curves stratified by Her2 expression levels in the Training Set.

The multivariate Cox regression revealed that postoperative pathological T staging (T3+T4,HR = 3.434,95%CI:1.975-5.97,P < 0.001), postoperative pathological N staging (N1,HR = 3.204,95%CI:2.101-4.885,P < 0.001; N2,HR = 5.284,95%CI:2.507-11.139,P < 0.001; N3,HR = 7.587,95%CI:4.381-13.14,P < 0.001),sTIL expression levels (sTIL intermediate expression, HR = 0.408, 95% CI: 0.275-0.604,P < 0.001; sTIL high expression, HR = 0.044, 95% CI: 0.021-0.091,P < 0.001), and Her2 expression levels (IHC 2+/Fish-, HR = 2.392, 95% CI: 1.513-3.782,P < 0.001) were independent predictive factors for DFS in premenopausal TNBC patients (Table 2). A nomogram integrating these variables was constructed to estimate 1-, 3-, and 5-year DFS probabilities (Figure 2). The model exhibited excellent discrimination, with C-indices of 0.862 (training) and 0.861 (validation). Time-dependent receiver operating characteristic (ROC) analysis further confirmed its robust predictive accuracy, with area under the curve (AUC) values for 3-year DFS of 0.907 and 0.908 in the training and validation sets, respectively (Figures 3, 4). Calibration curves demonstrated close alignment between predicted and observed outcomes across all time points (Figures 5–10). Kaplan-Meier analyses visually affirmed the prognostic stratification afforded by each variable (Figures 11–14).

**TABLE 2:** COX Univariate and Multivariate Analysis of Disease-free survival for TNBC patients.

Variables	Univariate analyses			Multivariate analyses		
	HR	95%CI	P	HR	95%CI	P
Age at diagnosis						
<50 years	1	​	​	1	​	​
≥50 years	0.445	0.246–0.807	0.008	0.605	0.326–1.124	0.112
Family history						
No	1	​	​	—	—	—
Yes	0.586	0.323–1.062	0.078	—	—	—
Surgical approach						
Radical surgery	1	​	​	—	—	—
Breast-conserving surgery	0.964	0.644–1.445	0.86	—	—	—
Postoperative pathological T staging						
T1	1	​	​	1	​	​
T2	1.6	0.985–2.600	0.058	1.623	0.969–2.719	0.066
T3+T4	4.264	2.596–7.004	<0.001	3.434	1.975–5.97	<0.001
Postoperative pathological N staging						
N0	1	​	​	1	​	​
N1	3.882	2.609–5.776	<0.001	3.204	2.101–4.885	<0.001
N2	2.967	1.462–6.021	0.003	5.284	2.507–11.139	<0.001
N3	19.799	11.683–33.554	<0.001	7.587	4.381–13.14	<0.001
Tumor grade						
G1+G2	1	​	​	1	​	​
G3	2.242	1.583–3.176	<0.001	1.341	0.923–1.948	0.123
Lymph-vascular invasion						
No	1	​	​	—	—	—
Yes	1	0.707–1.414	1	—	—	—
sTIL expression levels						
Low	1	​	​	1	​	​
Intermediate	0.494	0.344–0.710	<0.001	0.408	0.275–0.604	<0.001
High	0.041	0.021–0.083	<0.001	0.044	0.021–0.091	<0.001
Her2 expression levels						
IHC 0	1	​	​	1	​	​
IHC 1+	1.695	1.074–2.674	0.023	1.177	0.734–1.887	0.499
IHC 2+/Fish-	2.298	1.488–3.549	<0.001	2.392	1.513–3.782	<0.001
Ki67						
Ki67 ≤ 20	1	​	​	1	​	​
Ki67 > 20	0.326	0.184–0.579	<0.001	0.759	0.384–1.498	0.426
CK5/6						
Negative	1	​	​	—	—	—
Positive	0.813	0.543–1.219	0.317	—	—	—
EGFR						
Negative	1	​	​	—	—	—
Positive	1.088	0.675–1.754	0.729	—	—	—
Radiation therapy status						
No	1	​	​	—	—	—
Yes	1.004	0.648–1.554	0.987	—	—	—

## 4 Discussion

In this large, multicentre retrospective study, we developed and validated a prognostic nomogram specifically tailored for premenopausal women with TNBC receiving anthracycline–taxane chemotherapy. The model-incorporating T stage, N stage, sTIL density, and HER2 immunohistochemistry (IHC) status-demonstrated high predictive accuracy and clinical utility, addressing a critical unmet need in this high-risk population.

Our findings confirm the prognostic relevance of the tumour immune microenvironment in this cohort. Low sTIL density was independently associated with worse disease-free survival, corroborating previous studies that have consistently linked lower sTIL levels with inferior outcomes in patients treated with DNA-damaging chemotherapies (M Geurts et al., 2024; Denkert et al., 2018; Adams et al., 2019). Clinically, these results align with the concept that tumours with low sTILs frequently exhibit an immunologically “cold” phenotype, which may inform future therapeutic strategies aimed at enhancing antitumour immunity in such patients (Sharma et al., 2017; Emens and Middleton, 2015).

We also observed that HER2 IHC 2+/fluorescence *in situ* hybridization (FISH)-negative status—but not IHC 1+—independently predicted poorer disease-free survival, challenging several earlier reports that associated HER2-zero expression with inferior outcomes in TNBC (Miglietta et al., 2025; Won and Spruck, 2020). This discrepancy may reflect variations in patient selection, treatment setting, or HER2 scoring methodology. Multivariable analysis under the standard HER2-low definition (IHC 1+ or IHC 2+/FISH-negative) indicated that the prognostic risk was predominantly driven by the IHC 2+/FISH-negative subgroup. This suggests that HER2-low TNBC may not represent a homogeneous entity and that IHC 2+/FISH-negative tumours could harbour distinct biological properties—a hypothesis that warrants further validation.

Growing preclinical and clinical evidence indicates that HER2-low breast cancers exhibit altered receptor trafficking, activation of compensatory signalling pathways (e.g., PI3K/AKT, MAPK), and specific tumour–stroma interactions, which may collectively compromise chemotherapy efficacy (Tarantino et al., 2020; Schettini et al., 2021; Modi et al., 2022). Translationaly, the recent efficacy of novel antibody–drug conjugates such as trastuzumab deruxtecan in HER2-low metastatic breast cancer underscores the clinical relevance of this subset and highlights its potential susceptibility to targeted approaches (Banerji et al., 2019; Gennari et al., 2021). Our data suggest that premenopausal patients with HER2-low TNBC—particularly those with IHC 2+/FISH-negative disease—represent a candidate population for adjuvant antibody–drug conjugate trials. Such a strategy could eventually shift the therapeutic paradigm from empirical chemotherapy toward biomarker-guided targeted treatment.

Moreover, TNBC is a molecularly heterogeneous disease, encompassing distinct subtypes such as those described by Lehmann BD et al. (basal-like, immunomodulatory, mesenchymal, etc.) (Lehmann et al., 2011). The prognostic role of sTILs and HER2-low status may vary across these subtypes, a nuance not captured in our retrospective analysis. Future prospective studies integrating transcriptomic subtyping with tumour microenvironment features are warranted to refine personalised prognostic models.

By restricting our analysis to premenopausal women, we minimised confounding from age-related biological differences, such as the higher prevalence of PIK3CA mutations in older patients (Ciriello et al., 2015; Cancer Genome Atlas Network, 2012). This enhances the biological coherence and clinical applicability of our model for this specific demographic. The nomogram’s performance (C-index >0.86) surpasses that of many conventional prognostic tools and genomic assays (Paik et al., 2004; Cardoso et al., 2019), supporting its utility in routine practice for risk-adapted therapeutic decision-making.

From a translational perspective, our model provides a rationale for personalised adjuvant strategies. High-risk patients-those with low sTILs and HER2-low tumours-may benefit from treatment intensification, including capecitabine maintenance (Wang et al., 2021; Masuda et al., 2017) or PARP inhibition in BRCA-mutated cases (J Tutt et al., 2021; Robson et al., 2017). Conversely, low-risk patients could be spared unnecessary toxicity. Furthermore, this tool may facilitate refined patient selection for clinical trials evaluating immunomodulatory agents or novel antibody–drug conjugates in the early-stage setting.

## 5 Limitations

Several limitations of our study warrant consideration. First, its retrospective design introduces potential selection bias, despite multicentre enrolment. Second, all participants were of Chinese ancestry; external validation in diverse ethnic and geographic populations is essential to confirm generalisability (Eniu et al., 2008). Third, although patients were randomly assigned to training and validation sets, some baseline variables (e.g., family history, postoperative pathological T staging, Ki-67 index, and EGFR status) showed uneven distributions. While our model maintained high predictive performance across cohorts, such imbalances could theoretically introduce selection bias or affect generalizability. Future validation in larger, multi-ethnic cohorts is warranted to confirm the model’s stability. Fourth, although HER2 and sTIL assessments followed international guidelines and underwent central review, HER2 scoring reproducibility remains a challenge in TNBC, and sTIL evaluation is semi-quantitative. Future iterations could benefit from digital pathology, automated image analysis, or standardized immune-based assays to improve objectivity and reproducibility (Klauschen et al., 2018; Vanguri et al., 2022). Fifth, we did not incorporate germline BRCA status, a known determinant of TNBC prognosis and treatment response (Tutt et al., 2010; Tung et al., 2020). Integrating genomic and transcriptomic data in future prospective studies may further refine predictive accuracy and biological insight (Curtis et al., 2012; Pereira et al., 2016).

## 6 Conclusion

In conclusion, we have identified and validated low stromal TILs and HER2-low status as key biomarkers of intrinsic resistance to anthracycline-taxane chemotherapy in premenopausal TNBC. Beyond prognostication, these factors illuminate distinct biological pathways underlying treatment failure-an immunosuppressive microenvironment and the unique biology of HER2-low tumors. This mechanistic insight provides a compelling rationale for future pharmacological strategies: leveraging immunotherapy to inflame sTIL-low tumors and deploying novel ADCs to target HER2-low subtypes. Our findings thus offer a practical framework for patient stratification and pave the way for more personalized, mechanism-driven adjuvant therapy trials in this challenging disease.

## Data Availability

The original contributions presented in the study are included in the article/supplementary material, further inquiries can be directed to the corresponding authors.
